# Changes in the skin microbiome associated with squamous cell carcinoma in transplant recipients

**DOI:** 10.1038/s43705-022-00095-7

**Published:** 2022-02-01

**Authors:** Annika Krueger, Julian Zaugg, Nancy Lachner, Seweryn Bialasiewicz, Lynlee L. Lin, Sharon Gabizon, Priyamvada Sobarun, Mark Morrison, H. Peter Soyer, Philip Hugenholtz, Ian H. Frazer

**Affiliations:** 1 The University of Queensland Diamantina Institute, Faculty of Medicine, University of Queensland, Translational Research Institute, Woolloongabba, QLD Australia; 2 Australian Centre for Ecogenomics, University of Queensland, St Lucia, QLD Australia; 3 Queensland Paediatric Infectious Diseases Laboratory, Children’s Health Queensland, South Brisbane, QLD Australia; 4 The University of Queensland Diamantina Institute, The University of Queensland, Dermatology Research Centre, Brisbane, QLD Australia; 5 Dermatology Department, Princess Alexandra Hospital, Brisbane, QLD Australia

**Keywords:** Clinical microbiology, Bacterial genetics

## Abstract

Actinic keratoses (AK) arise in severely photo-damaged skin and can progress to squamous cell carcinomas (SCC). AK and SCC are common in Caucasian populations, and immunosuppressed individuals have a markedly higher risk of developing SCC. An overabundance of *Staphylococcus aureus* has been reported in AK and SCC lesions of immunocompetent individuals, however, the AK/SCC microbiome in immunosuppressed cohorts has not been investigated. Here, the microbial profile and bacterial load of AK, SCC and control skin swabs from 32 immunosuppressed organ transplant recipients were characterised via SSU rRNA gene sequencing and qPCR, and compared to a previously described immunocompetent cohort. Although the taxonomic composition of skin swab samples was mostly subject-specific, significant differences were observed between control skin, AK, and SCC in both cohorts. Surface bacterial load was increased and alpha diversity decreased in AK and SCC compared to control skin due to an increased abundance of *Staphylococcus* species and relative decrease of skin commensals. *Staphylococcus epidermidis* predominated on SCC from transplant recipients in contrast to SCC of immunocompetent subjects dominated by *S. aureus*. In conclusion, AK and SCC of immunosuppressed and immunocompetent subjects present with distinctive microbial dysbioses, which may be relevant to SCC pathogenesis and progression.

## Introduction

Keratinocyte cancers are the most common cancer in Caucasian populations worldwide [1]. Though squamous cell carcinomas (SCC) are a less prevalent type of keratinocyte cancer than basal cell carcinoma, SCC more commonly metastasise resulting in a greater mortality rate [2]. SCC typically arise from pre-malignant lesions termed actinic keratoses (AK). Individuals receiving immunosuppressive medication, including organ transplant recipients, have a higher prevalence of AK and SCC and an associated increased risk of metastasis and death [3, 4]. Other predominant risk factors include UV irradiation, advancing age, light skin colour and chronic inflammation [1]. An infectious aetiology has also been suspected to play a role in skin malignancy [5–7]. Individuals with an impaired immune function are more susceptible to infection, which may increase their risk of microbially linked cancers [7].

To date, there is no clear consensus regarding the role of microorganisms in SCC development. In immunocompetent cohorts, both AK and SCC have been associated with an increased prevalence of β-human papillomavirus and *Staphylococcus aureus* [5, 6, 8–10]. We previously conducted a longitudinal and cross-sectional study investigating the microbial profiles of AK and SCC skin swabs from 13 immunocompetent men. This study showed a reduction of commensal microorganisms, such as members of the genus *Malassezia* and *Cutibacterium*, and increase in relative abundance of *S. aureus* in AK and SCC samples, compared to the control skin swabs [10]. To our knowledge, a comprehensive study investigating the microbiome associated with AK and SCC in immunosuppressed individuals has not been performed.

Here, we describe the skin microbiomes of normal and photo-damaged skin (PDS), AK, and intraepidermal and invasive SCC, from a cohort of 32 immunosuppressed organ transplant recipients. For comparison, we reanalysed data from our previous study of 13 immunocompetent subjects [10] and, additionally, assessed the bacterial load of control, AK and SCC skin swabs from these subjects. Consistent with previous studies [5, 6, 10], we report an increased abundance of staphylococci in AK and SCC. Whereas we and others observed an overabundance of *S. aureus* on AK and SCC from immunocompetent individuals, *Staphylococcus epidermidis* predominated on SCC lesions from transplant recipients. In both cohorts, a reduction in overall local species diversity, and a progressively increased absolute bacterial load was noted in AK and SCC when compared to control skin. Overall, our findings highlight microbial dysbiosis as a common feature of AK and SCC lesions.

## Materials and methods

A detailed version of the materials and methods can be found in the supplements.

### Ethics, subjects and sampling

Thirty-two organ transplant recipients receiving long-term immunosuppressive medication were recruited at the Princess Alexandra Hospital (Brisbane, Australia) under an ethically approved protocol (HREC/11/QPAH/477). Enroled were male and female subjects aged 44–80 who were at least one year post-transplant and presenting with AK and/or intraepidermal/invasive SCC (subject demographics in Supplementary Table S1; clinical and histopathological features of sampled AK/SCC in Supplementary Fig. S1). Subjects gave their written, informed consent. Exclusion criteria were the presence of chronic skin disorders, and current or prior use (in the preceding three months) of antibiotics and/or topical agents to treat AK. Skin swab samples were collected from PDS and AK on the forearm. SCC and matching perilesional controls (SCC_PL) were sampled from the forearms as well as other body sites. Nine of the subjects were age-matched transplant recipients without AK/SCC and mild photo-damage. Skin swabs from the forearms of these subjects served as normal skin (NS) controls. Control swabs from non-exposed areas were not obtained as part of this study.

Sterile swabs were dipped into sterile 0.15 M sodium chloride solution and firmly rotated over a ~1.5 cm^2^ sampling area for 30 s. Swabs were collected in sterile glycerol-saline solution and DNA extraction buffer and stored immediately at −80 °C. Negative control swabs not brought into contact with skin were collected in each sampling session and processed identically to skin sample swabs. In total, 216 swab samples (including 19 negative controls) from transplant recipients were processed for SSU rRNA gene amplicon sequencing.

SSU rRNA gene amplicon data from our previous longitudinal study [10] on subjects without known systemic immunosuppression was used for comparative analyses. This cohort was described as ‘immunocompetent’ although it should be noted that extensive UV exposure can downregulate immunity in seemingly immunocompetent individuals [11]. Details on study design and demographics for this cohort can be found in Wood et al. [10] and in Supplementary Table S2. Two hundred fifty-seven samples (samples from one visit per subject, including 33 negative controls) collected in this previous study were included to match the cross-sectional study design.

### SSU rRNA gene amplicon profiling

DNA extraction and amplicon sequencing was performed as described previously [10]. In brief, DNA was extracted with the PowerSoil DNA isolation kit (Qiagen) and the SSU rRNA genes were PCR amplified with universally conserved primers (926F/1392R). PCR amplicons were purified, indexed with barcodes, and sequenced on the MiSeq system (Illumina).

Primer sequences were removed from forward and reverse de-multiplexed reads using cutadapt (ver. 2.4) [12], with reads not containing primers discarded. Using QIIME2 (ver. 2020.8.0) [13], paired reads were filtered, dereplicated, merged and chimeras removed by DADA2 (--p-trunc-len-f 270 and --p-trunc-len-r 230) [14]. Taxonomy was assigned to the resulting amplicon sequence variants (ASV) [15] by aligning each (classify-consensus-blast) against the non-redundant SILVA database (release 138) [16].

### Amplicon data analysis and statistics

Amplicon data analyses were performed in R (ver. 4.0.2). ASVs that were not bacterial, fungal or archaeal in origin, classified at below the phylum level, or that were classified as chloroplast or mitochondria, were discarded. For each cohort, the decontam package (ver. 1.8.0; method = prevalence, threshold = 0.5) [17] was used to identify and remove likely contaminant ASVs that were more prevalent in negative control swabs (Supplementary Figure S2). ASVs with a relative abundance less than 0.05% in non-negative samples were also removed, and samples below 2,000 reads discarded. After removal of low depth samples, 182 transplant recipient (25 NS, 72 PDS, 42 AK, 22 SCC_PL, 21 SCC) and 206 immunocompetent (50 PDS, 87 AK, 34 SCC_PL, 35 SCC) samples remained for further analysis. Sample depth was limited to a maximum of 30,000 reads by using the rrarefy function in vegan (ver. 2.5-7) [18].

ASV counts were collapsed to the genus level and transformed to centred log-ratio [19] values prior to principal component analysis (PCA) and permutational multivariate analysis of variance (PERMANOVA) [20]. The rda function from vegan was used to perform PCA and figures were created with base R graphics and vegan functions. PERMANOVA was performed using the adonis function from vegan with Euclidean distances and 999 permutations.

Alpha diversity metrics Chao1 (richness), Shannon (diversity) and Simpson (evenness) were calculated using phyloseq (ver. 1.32.0) at the genus level on samples rarefied to 5000 reads [21]. Significant differences in alpha diversity distributions were determined either through Kruskal–Wallis tests followed by Benjamini & Hochberg [22] corrected Dunn’s multiple comparisons tests or Mann–Whitney *U* tests.

Differentially abundant genera and ASVs with ≥30 reads in at least one sample were identified using the Wald test in DESeq2 [23] (ver. 1.28.1; fitType = parametric), with *p* values corrected for multiple testing using the Benjamini and Hochberg method [23]. DESeq2 fits count data to a negative binomial generalised linear model and tests for significant differences between groups. In the present study, each sample type was compared within each cohort. In addition, male and female microbiomes were compared for each sample type in the transplant recipient cohort. Result tables were extracted using the results function in DESeq2 with both independent filtering and Cooks cut-off set to false and alpha set to 0.05. Complementary to DESeq2, differentially abundant genera were also identified by comparing relative abundance distributions: for each cohort, the top 30 genera by mean relative abundance for each sample type were identified and relative abundance distributions compared with Kruskal–Wallis tests followed by Benjamini & Hochberg corrected Dunn’s multiple comparisons tests. The relative abundances of the top five genera according to duration of immunosuppression—2 to 6, 7 to 15, and 16 and higher (years)—for each sample type, were compared in the same manner.

Boxplots, stacked bar charts and scatter plots were created with ggplot2 (ver. 3.3.3) [24]. Heatmaps were created using ComplexHeatmap (ver. 2.4.3) [25]. The cowplot (ver. 1.1.1) and lemon (ver. 0.4.5) packages were used for the formatting of figures.

### Isolation and genome sequencing of staphylococci

Viable microbes sampled by swabbing were preserved in glycerol-saline solution at −80 °C. SCC and SCC_PL samples (*n* = 24) from transplant recipients were plated onto staphylococci-selective mannitol salt agar. After 48 h at 37 °C, colonies were assessed. A representative sample of each colony type (based on colour, size, morphology, ability to ferment mannitol) from each SCC sample were subcultured. From overnight liquid cultures of single colonies, bacteria were harvested by centrifugation, resuspended in DNA extraction buffer and stored at −80 °C until further processing for draft genome sequencing. The detailed procedures for DNA extraction, purification, library preparation and sequencing are provided in the supplements.

Raw reads for isolated strains were processed with Trimmomatic (ver. 0.39; LEADING:3, TRAILING:3, SLIDINGWINDOW:4:15, MINLEN:50) for quality filtering [26]. Quality filtered reads were then assembled using SPAdes (ver. 3.14.0) [27] as part of the Shovill assembly pipeline (ver. 1.1.0, T. Seeman, unpublished, https://github.com/tseemann/shovill; --opts “--isolate”, --minlen 1000), with the completeness and contamination of each assembly evaluated using CheckM (ver. 1.1.2) [28] and taxonomy assigned using the Genome Taxonomy Database Toolkit (GTDB-Tk, ver. 1.6) with reference to the GTDB database release R06-R202 [29, 30].

### 6-HAP marker and *gseA* gene analysis of staphylococci genomes

To determine the presence of 6-N-hydroxyaminopurine (6-HAP) marker genes in *S. epidermidis* isolated from transplant recipient SCC, previously published methods were followed [31]. Briefly, reads for each isolate were mapped to the 28 marker genes using Bowtie 2 (ver. 2.2.9; --very-sensitive) [32], with the coverage of each gene calculated using samtools (ver. 1.8) [33]. A gene was considered present if it had at least 95% coverage. To evaluate the prevalence of 6-HAP marker genes among a broader selection of *Staphylococcus* genomes, gene sequences were also aligned against 11,300 RefSeq and GenBank genomes classified as *Staphylococcus* according to the Genome Taxonomy Database [34], and the 6-HAP producing *S. epidermidis* MO34 strain (Genbank assembly accession GCA_009932695.1, https://www.ncbi.nlm.nih.gov/assembly/GCF_009932695.1/) [31], using blast+ [35]. A gene was considered present when 90% of its sequence was aligned with a 95% sequence identity.

To determine the presence of the glutamyl endopeptidase gene (*gseA*), translated coding sequences from all isolates were predicted by Prodigal [36] (ver. 2.6.4) as part of Prokka [37] (ver. 1.14.6) and aligned against a reference *gseA* sequence (https://www.uniprot.org/uniprot/P0C0Q1) with blast+ [35]. The gene was considered present when 90% of its sequence was aligned with a 95% sequence identity.

### Phylogenetic and virulence gene analysis of staphylococci genomes

The pathogenicity of the 61 *S. epidermidis* isolate draft genomes, and 17 reference genomes (Supplementary Table S3), was evaluated by screening for the presence of antimicrobial resistance and virulence genes with ABRicate (ver. 1.0.1, https://github.com/tseemann/-ABRICATE), using the Comprehensive Antibiotic Resistance Database (CARD) [38], Virulence Factor Database (VFDB) [39] and ResFinder database [40]. Gene protein sequences were also aligned against reported *S. epidermidis* virulence factors [41] (Supplementary Table S3) through a blastp [35] search. As short peptide sequences can often be missed during genome annotation [42], the *S. epidermidis* virulence factors were also aligned against the isolate and reference genomes through a tblastn search [35]. For both blast searches, genes were considered present when at least 70% of the reference sequence aligned with 70% identity.

A phylogenetic tree was constructed for the *S. epidermidis* isolate draft and reference genomes, based on a core-alignment of single nucleotide polymorphisms followed by the removal of recombinant regions. Genomes were aligned against the *S. epidermidis* GTDB representative genome NBRC 100911 (Genbank accession GCA_006742205.1) using Parsnp (ver. 1.5.6) [43], with recombinant regions identified and removed using Gubbins (ver. 3.0.0) [44]. A maximum-likelihood phylogenetic tree was constructed using RAxML (ver. 8.2.12) using a general time-reversible nucleotide substitution model with gamma correction for site variation (GTRGAMMA) and 1000 bootstraps.

To evaluate the relatedness of all staphylococci isolates, a pangenome analysis was performed with Panaroo (ver. 1.2.8; --clean-mode strict, --core_threshold 0.90, --threshold 0.95, --alignment core) [45] on the 113 draft genome sequences (108 organ transplant recipient, 5 immunosuppressed) and 31 reference genomes (representative/type strain for each species and other clinically relevant strains; Supplementary Table S3). A maximum-likelihood phylogenetic tree was built from the resulting core gene alignment using IQ-Tree (ver. 2.1.2, -m GTR). Phylogenetic trees and virulence gene presence/absences were visualised using the R packages ggtree (ver. 3.0.4) [46], ggplot2 [24] and cowplot.

### Quantification of total DNA and bacterial load

A subsample of collected swabs was tested in duplicate for total DNA content using the Quant-iT™ dsDNA Assay Kit (#Q33120) (ThermoFisher Scientific, Australia). qPCR was performed on all swab samples using bacteria-specific SSU rRNA primers (1406F/1525R). The PCR was prepared using 5 µL of 2X QuantiNova SYBR Green PCR Kit (Qiagen, Germany), 4 µL of skin swab DNA and 1 µL of primer mix. Samples were run in triplicate on the ViiA7 platform (Applied Biosystems, USA). Cycling conditions and preparation of the quantification standard are described in the supplements. Bacterial SSU rRNA gene copy numbers were calculated using QuantStudio Real-Time PCR Software v1.3.

### *Staphylococcus* and *Staphylococcus aureus* duplex qPCR

To estimate *Staphylococcus* and *S. aureus* loads in skin swabs, a duplex real-time PCR assay was adapted from Kilic et al. [47] and is described in detail in the supplemental methods. In short, five replicates of each swab sample were assessed via probe-based qPCR assay targeting the *tuf* (*Staphylococcus* genus) and *nuc* (*S. aureus-*specific) gene. Genome copy equivalents (Geq/µL) were calculated based on a quantification standard. The limit of confident detection was determined from 20-replicate twofold dilutions and defined as the lowest concentration able to be detected in 95% of replicates [48]. The limit of quantification was defined as the lowest standard concentration with <35% coefficient of variation [48]. GraphPad Prism (ver. 8.3.1) was used for statistics and visualisation of the qPCR data.

### Deferred growth inhibition (competition) assay

Twelve SCC-associated *S. epidermidis* isolates, each from a different transplant recipient, and reference strain *S. epidermidis* ATCC14990, were inoculated in tryptic soy broth (TSB) and incubated overnight at 37 °C, 250 rpm. The next morning, each *S. epidermidis* culture was diluted to an OD_600_ of 0.1 in 1 ml TSB, and 10 µL of *S. epidermidis* dilution was carefully pipetted as a spot culture onto a tryptic soy agar (TSA) and moved to the 37 °C incubator. On the same day, six genetically distinct *S. aureus* clinical isolates as well as the methicillin-resistant *S. aureus* USA300 were inoculated in TSB and placed in an orbital shaker at 37 °C and 250 rpm. The following day, each *S. aureus* culture was diluted to an OD_600_ of 0.07 in 50 ml TSA (0.6% agar) and carefully pipetted to cover the agar surface of the plates prepared the previous day containing *S. epidermidis* spot culture. After 24 h incubation at 37 °C, plate images were taken, and the growth inhibition zone (visible zone of no or reduced growth of *S. aureus*) was measured in millimetres using ImageJ. At least three experimental replicates of each *S. aureus*-*S. epidermidis* combination were obtained.

## Results

### Study cohorts and sample collection

A total of 216 skin swabs were collected from a cohort of 32 organ transplant recipients who had been on immunosuppressive medication for at least one year (28 ≥ 5 years; 16 ≥ 15 years; see Supplementary Table S1 for detailed subject demographics). Recruited subjects had an average age of 62 years and were mostly Caucasian males with classical ‘field cancerisation’ on their forearms, clinically characterised as a thickened, leathery skin with fine scales and mottled pigmentation (Supplementary Fig. S1). Pre-malignant AK (*n* = 50) and non-malignant PDS (*n* = 73) were exclusively sampled from the forearm, which was the focal area of our study as high UV exposure to that region is expected. Due to their low occurrence, malignant intraepidermal (*n* = 11) and invasive (*n* = 13) SCC samples were obtained from the forearm as well as other body sites (see Supplementary Table S1 for SCC locations). Matching each SCC sample, site-specific perilesional control swabs were also collected. The forearms of nine age-matched transplant recipients with mild photo-damage and no AK or SCC were sampled as NS controls (*n* = 27).

For comparison, a subset of swabs from PDS, AK and SCC collected as part of a previously published longitudinal study of immunocompetent subjects (13 Caucasian males, with an average age of 72 years) [10] were included and further analysed in this study. The cross-sectional design of the current study was matched by subsampling the previous longitudinal dataset to include only skin swabs from a single visit per subject (257 swabs; see Supplementary Table S2). Subsampled swabs included 55 PDS, 95 AK, 39 SCC and 35 site-specific perilesional controls. Note that the study of immunocompetent subjects lacked a control group, i.e., NS control swabs from individuals with mild photo-damage and no prior or current AK or SCC.

### Skin microbiome relative abundance profiles

**Fig. 1:**
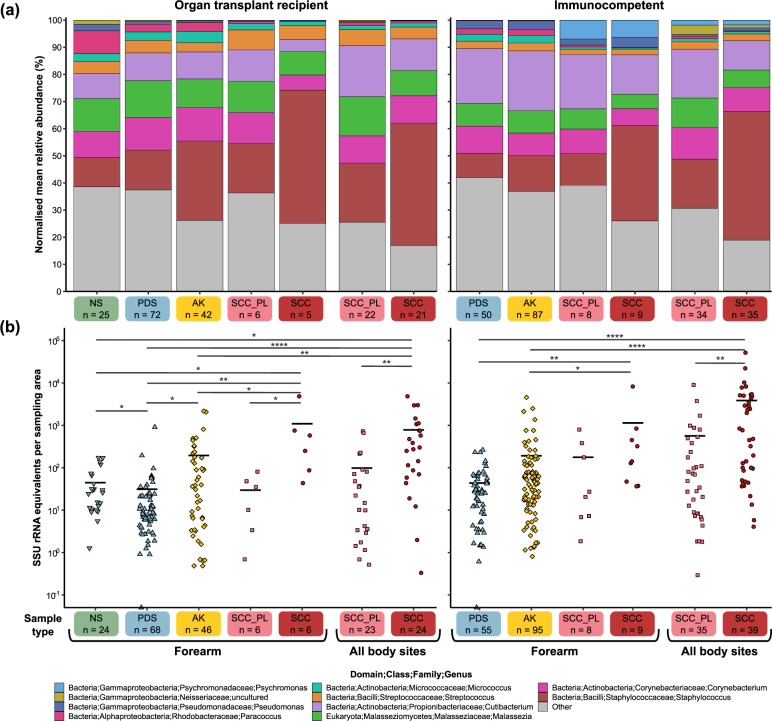
Relative microbial abundances and absolute bacterial load in AK, SCC and control skin of immunosuppressed and –competent subjects. **a** Normalised mean relative abundances of the five most abundant skin microbes in swabs from normal skin (NS), non-malignant photo-damaged skin (PDS), actinic keratosis (AK), intraepidermal and invasive squamous cell carcinoma (SCC) and matching perilesional controls (SCC_PL) from organ transplant recipient and immunocompetent cohorts. Microbes with lower abundances have been combined into ‘Other’ (in grey). Values are calculated from normalised SSU rRNA read counts collapsed to the genus level. **b** Bacterial load in skin swab samples from organ transplant recipients and immunocompetent subjects assessed via SSU rRNA qPCR. The mean is represented by the black bar and significant differences between sample types, as calculated by Dunn’s multiple comparisons tests, are indicated by *p ≤ 0.05, **p ≤ 0.01, ***p ≤ 0.001 and ****p ≤ 0.0001.

**Fig. 2:**
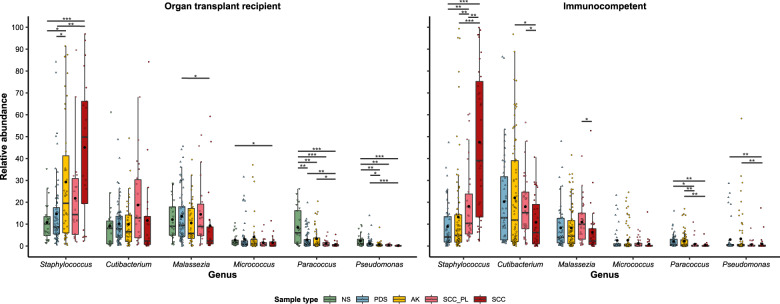
Relative abundance of the *Staphylococcus* genus and other skin commensals in swab samples from organ transplant recipients and immunocompetent subjects. Tukey style box plots showing the relative abundances (normalised SSU rRNA read counts) of the *Staphylococcus* genus and other skin commensals in swab samples from organ transplant recipients and immunocompetent subjects. Bars indicate the median ± 1.5 × interquartile range and the mean relative abundance for each sample type is indicated by the black dot. Significant differences between sample types, calculated by Dunn’s multiple comparisons tests, are indicated by *p ≤ 0.05, **p ≤ 0.01 and ***p ≤ 0.001.

**Fig. 3:**
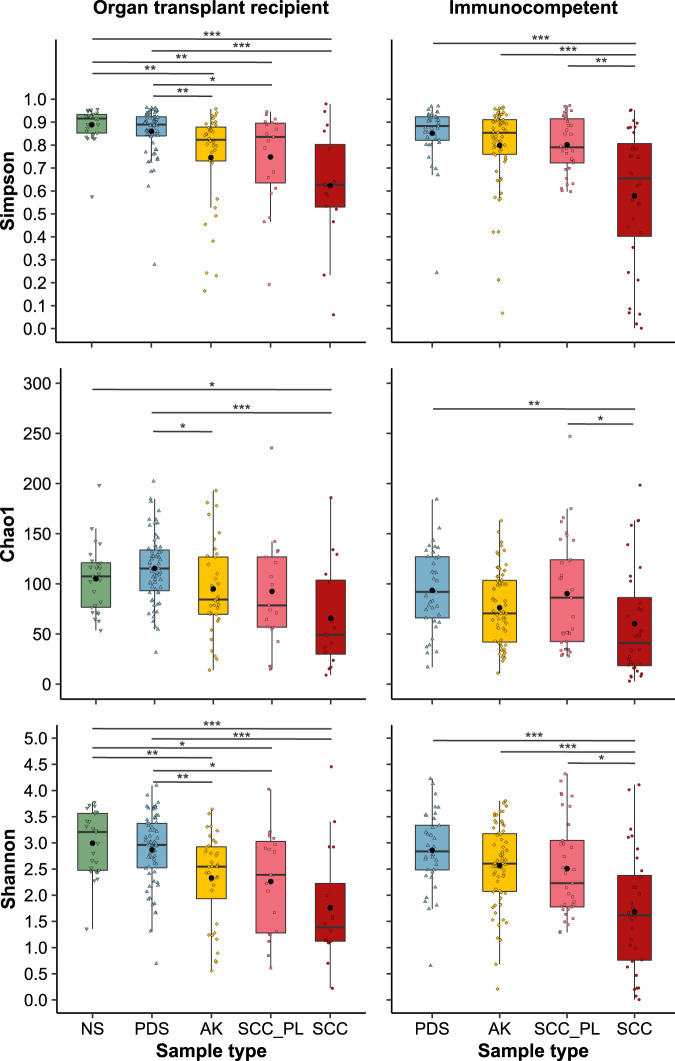
Alpha diversity in control skin, AK and SCC swabs from immunosuppressed and immunocompetent individuals. Tukey style box plots showing genus diversity across sample types for organ transplant recipient and immunocompetent cohorts. Diversity is represented by three separate measures of alpha diversity; Chao1 (richness), Shannon (diversity) and Simpson (evenness). Bars indicate median ± 1.5 × interquartile range and the mean diversity for each sample type is indicated by the black dot. Significant differences between sample types are indicated by *p ≤ 0.05, **p ≤ 0.01 and ***p ≤ 0.001 as calculated via Dunn’s multiple comparisons test.

Small subunit ribosomal RNA (SSU rRNA) gene amplicon sequencing was performed to characterise the microbiome profiles of the collected skin swab samples. A universally conserved region was targeted to capture most bacteria, archaea and eukaryotes. PCA and non-parametric multivariate statistical testing (PERMANOVA) showed that the variance in microbial composition between samples was predominantly attributable to inter-individual differences (*p* = 0.001), however, the different sample types (i.e. controls, AK, SCC) also contributed a statistically significant amount of variation (*p* = 0.001) in both cohorts (Supplementary Figs. S3, S4 and Supplementary Table S5). The most evident difference in microbial composition between sample types was the increasing relative abundance of the genus *Staphylococcus* from control skin to AK to SCC (Figs. 1a, 2 and Supplementary Figs. S5–S9). On the forearm of transplant recipients, NS samples had a *Staphylococcus* mean relative abundance of 10.8%, PDS 14.7%, and AK 29.3% (vs NS *p* = 0.034 vs PDS *p* = 0.029) (Fig. 2). The highest mean relative abundance was observed on SCC (45.1%), which presents a 2-fold increase compared to perilesional controls (21.8%). Similarly, in the immunocompetent cohort, the *Staphylococcus* mean relative abundance was 8.9% in PDS, 13.2% in AK and 47.5% in SCC, with a 2.6-fold increase in mean relative abundance between SCC and perilesional skin (*p* = 0.008). The increasing *Staphylococcus* relative abundance was accompanied by a significant reduction in the abundance of one or more skin commensals, including *Micrococcus, Paracoccus, Pseudomonas, Cutibacterium*, and *Malassezia* (Fig. 1a, Fig. 2, Supplementary Table S6 and Supplementary Table S7). An exception was a number of lesion samples from sebaceous skin sites, such as the forehead, scalp and nose, which were overpopulated by *Cutibacterium* and/or *Malassezia*, lipophilic microbes that typically thrive in areas with a high density of sebaceous glands [49] (Supplementary Figure S5). Alpha diversity significantly decreased with increasing lesion severity in both cohorts (Fig. 3). In the transplant recipient cohort, several ASVs for *Cutibacterium*, *Corynebacterium*, and *Staphylococcus* were found to be more abundant in males compared to females in PDS swab samples (*p* ≤ 0.001–0.05) (Supplementary Table S6), while female subjects had higher alpha diversities (*p* ≤ 0.001) (Supplementary Fig. S10). No consistent differences in community profiles were observed as a function of the length of immunosuppression (ranging from 2–40 years) (Supplementary Fig. S11, Supplementary Tables S6 and S7).

Skin in different anatomical locations harbour distinct microbiomes [49]. Whereas the NS, PDS and AK were exclusively sampled from the forearm, SCC and the matching perilesional swabs were obtained from the forearm and other body sites. To evaluate whether the sampling location influenced the relative abundance results, the SCC forearm samples were also analysed independently. In both cohorts, the mean relative abundance of the most abundant genera in forearm SCC and perilesional samples were comparable to those when all body sites were grouped (mean absolute difference < 3%) (Fig. 1a).

### Absolute bacterial abundances

As SSU rRNA gene amplicon sequencing does not provide information on the absolute abundance of microbes, we next quantified the bacterial load in skin swab samples from controls, AK and SCC. Extracted swab DNA from our previous study [10] had been stored at −80 °C and was available for further analysis. In both studies, skin swabs were collected by multiple clinicians allowing us to assess the effect of potential inter-operator differences in sample collection which could distort bacterial load measurements. No significant difference in total DNA concentrations were found between photo-damaged control swabs sampled by three different clinicians across both cohorts (mean DNA concentration: 0.039, 0.044 and 0.044 ng/μL; *p* = 0.46) (Supplementary Table S8). Quantitation of bacterial load in all samples was then assessed via bacteria-specific SSU rRNA qPCR. Compared to PDS, the average bacterial load per surface area of skin was 4–6-fold higher in AK and 25–88-fold higher in SCC in the transplant recipients and immunocompetent subjects, respectively (Fig. 1b). Higher bacterial loads in AK and SCC could also be observed at the individual subject level (Supplementary Figs. S12 and S13). Long-term immunosuppression (>6 years) appears to be associated with decreased bacterial load on all sample types (Supplementary Fig. S11).

### *Staphylococcus* abundance in skin swab samples

**Fig. 4:**
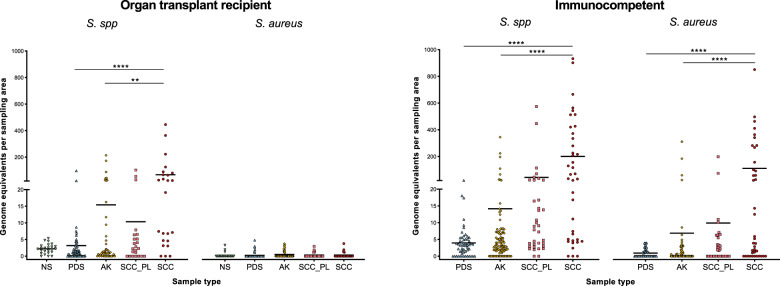
*Staphylococcus* species and *S. aureus* load in skin swab samples of organ transplant recipients and immunocompetent subjects. *Staphylococcus* species and *S. aureus* absolute abundance was determined by targeting the *tuf* and *nuc* gene in a duplex probe-based qPCR assay. Displayed is the mean. Statistically significant differences between sample types are indicated by *p ≤ 0.05, **p ≤ 0.01, ***p ≤ 0.001 and ****p ≤ 0.0001 as calculated via Dunn’s multiple comparisons test.

As *S. aureus* has been previously associated with SCC in immunocompetent cohorts [5, 6, 10], a duplex qPCR was performed to determine the abundance of this species in our samples in relation to other staphylococci. Consistent with the qualitative profiling results (Fig. 1a, b), the absolute abundance of *Staphylococcus* spp. was lowest in control skin, greater in AK and highest in SCC samples in both cohorts (Fig. 4). In the immunocompetent cohort, 28 of 39 SCC lesions had a strong positive signal for *S. aureus*, accounting on average for 57% of the total *Staphylococcus* abundance in these 28 lesions. On average, an 11.2-fold greater load of *S. aureus* was observed on SCC compared to perilesional control skin. In immunosuppressed transplant recipients, *Staphylococcus* spp. were 6.6-fold more abundant in SCC than in perilesional control skin, however, *S. aureus* was not detected in most samples (Fig. 4). Three of the SCC lesions had a weak positive signal for *S. aureus* that was below the confident qPCR detection limit.

Due to the unexpected low occurrence of *S. aureus* in swab samples from transplant recipients, and the limited resolution of bacterial species afforded by SSU rRNA gene amplicon sequencing [50], a selective culturing approach followed by genome sequencing was used to determine which *Staphylococcus* species dominated transplant recipient SCC. In line with our previous results, the majority of SCC swab samples (22 of 24), from 13 of the 14 transplant recipients with SCC, produced colonies on staphylococci-selective agar. By comparison, only 41% of the matching perilesional controls were positive on this medium, most of which had low colony counts. SCC typically produced high colony counts consistent with the observed high staphylococcal DNA loads (Fig. 4), confirming that detected bacterial DNA reflected viable bacteria. Eighty three percent of SCC had a ≥1000-fold increased number of colonies than their matching control (Supplementary Fig. S14). Single colonies were subcultured from 22 SCC samples and their genomes sequenced to enable species identification. From a total of 108 axenic isolates across the 13 transplant subjects, the most commonly identified species was *S. epidermidis* (*n* = 61; across 12 of 13 subjects; Table 1). Other less frequently isolated species included *S. schleiferi*, *S. capitis, S. haemolyticus*, *S. warneri* and *S. hominis*. Most lesions (72%) were dominated by a single *Staphylococcus* species, most commonly *S. epidermidis*, and only one was dominated by *S. aureus* consistent with a positive *S. aureus* qPCR signal for this sample. Pangenome analysis of staphylococci genomes indicated that strains clustered by species and subject (Supplementary Fig. S15).

**Table 1:** Staphylococcus species identified in SCC lesions from organ transplant recipients.

	No. of lesions colonised (of 22)	No. of subjects colonised (of 13)	Isolate no. in total (of 108)
*S. epidermidis*	16	12	61
*S. schleiferi*	3	1	14
*S. capitis*	3	2	10
*S. haemolyticus*	2	2	6
*S. warneri*	2	2	6
*S. hominis*	2	1	4
*S. aureus*	1	1	7

*Staphylococcus* species were isolated and identified by genome sequencing from 22 SCC lesions from 13 different organ transplant recipients. In total, 108 isolates were collected and characterised.

### Genetic and phenotypic characterisation of SCC-associated *S. epidermidis* isolates

**Fig. 5:**
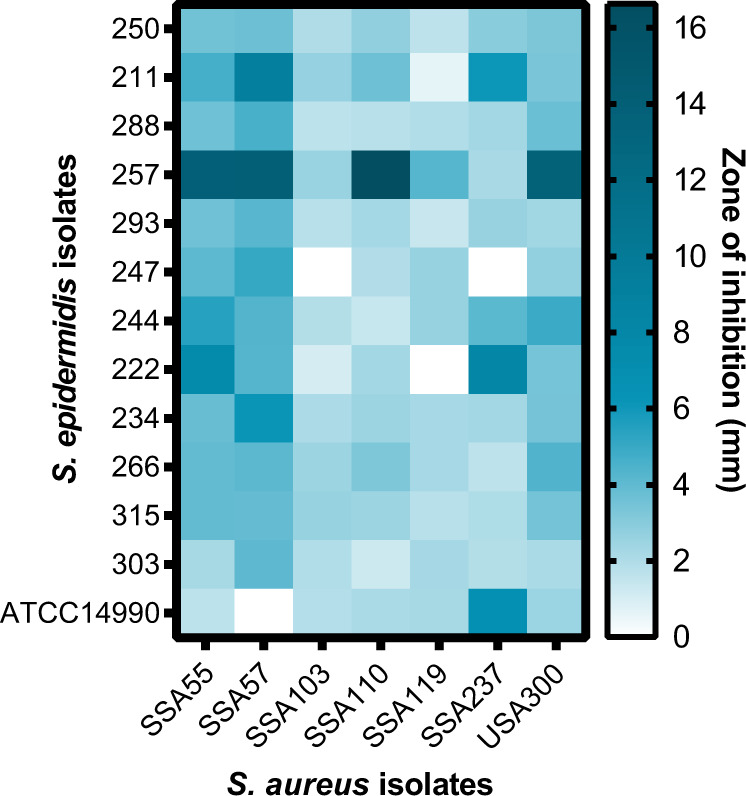
Deferred growth inhibition assay to quantify the inhibitory activity of *S. epidermidis* on *S. aureus* growth. Heatmap representing the size of the zone of inhibition (in mm) from competition assays of *S. epidermidis* isolates from transplant recipients and reference strain ATCC14990 versus *S. aureus* isolates and methicillin-resistant *S. aureus* USA300.

Unlike the observed association between *S. aureus* and SCC in immunocompetent cohorts, the culture and qPCR results indicated that coagulase-negative staphylococci (CoNS), and in particular *S. epidermidis*, predominated on SCC from transplant recipients (Table 1 and Fig. 4). *S. epidermidis* has been reported to actively inhibit *S. aureus* colonisation by secreting a serine protease, encoded by the gene *gseA* [51]. This gene appears to be common to the species (Supplementary Table S9), and was present in all 61 SCC-associated *S. epidermidis* isolate genomes (Supplementary Fig. S16). In line with this, bacterial competition experiments showed that 12 representative SCC-associated *S. epidermidis* isolates, each from a different transplant recipient, caused full and/or partial growth inhibition of six genetically distinct *S. aureus* and the community-associated methicillin-resistant USA300 strain in most cases (Fig. 5). The extent of inhibition varied, with some *S. epidermidis* isolates being particularly potent inhibitors, and with certain *S. aureus* isolates being more prone to inhibition than others. In addition to active inhibition, we evaluated if our *S. epidermidis* clinical isolates had a growth advantage provided by antibiotic resistance over antibiotic-sensitive *S. aureus*, by identifying the presence of antibiotic resistance genes (Supplementary Fig. S16). The *mecA, blaZ and dfrC* genes indicating oxacillin/methicillin, penicillin and trimethoprim resistance, respectively, were present in 57%, 84% and 92% of our *S. epidermidis* isolates. In addition, all clinical isolates were positive for the *fosB* and *norA* genes that mediate resistance to fosfomycin and quinolone antibiotics. Genes that are responsible for the resistance against other antibiotics, including erythromycin, tetracycline, fusidic acid, lincomycin, mupirocin, streptogramin A and gentamycin, were less common.

Interestingly, it has recently been proposed that *S. epidermidis* can be protective against skin cancer through the production of 6-HAP, a DNA polymerase inhibitor [31]. 28 6-HAP marker genes were identified in the same study as diagnostic of the protective phenotype. None of the 61 SCC-associated *S. epidermidis* isolate genomes obtained from transplant recipients contained the full set of 28 6-HAP marker genes. Notably, this was also the case for 556 *S. epidermidis* genomes sourced from public sequence databases (Supplementary Table S9).

Lastly, we carried out a virulence factor analysis to investigate the pathogenicity of SCC-associated *S. epidermidis* (Supplementary Figure S16). Virulence genes, including exoenzymes, lytic and pro-inflammatory peptide toxins, as well as factors that aid immune evasion and biofilm formation, were frequently present in the 61 SCC-associated *S. epidermidis* isolates.

## Discussion

Immunosuppression limits the hosts’ ability to detect and eliminate malignant cells [52] and thus presents a strong risk factor for the development of cancers including SCC [3, 4]. In addition, immunosuppressed individuals have a reduced capability to control and withstand microbial invasion and attack [52]. To identify potential microbial triggers for immunosuppression-associated SCC, this study evaluated the skin microbial profiles of SCC and precursor stages in a transplant recipient cohort. Like previous studies in immunocompetent cohorts [5, 10, 53], we found that compared to NS, AK and SCC of transplant recipients have a lower alpha diversity characterised by enrichment of the genus *Staphylococcus* and a relative reduction in commensal genera. The lower relative abundance of the lipophilic commensal species *Cutibacterium* and *Malassezia* on forearm lesions could be due to the scaly and dry environment typical of AK and SCC, thereby favouring the growth of dry-tolerant taxa such as *Staphylococcus*. Lesional skin was associated with a highly increased surface bacterial load. This may also be a consequence of the skin physiological changes underlying SCC formation. AK and SCC typically present with thickened and flaky skin, and a loss of intact skin surface, which may provide enhanced opportunities for microbial growth due to the increased colonisable surface area, and as the disrupted integrity of the skin allows bacteria to more easily adhere, obtain nutrients, and form biofilms [54]. The high bacterial load on skin lesions of immunosuppressed individuals has significant clinical implications as this may increase the likelihood of developing skin infections, bacteraemia and sepsis. Indeed, a large percentage (55–97%) of organ transplant recipients present with skin infections [55].

The present and previous studies highlight an association between skin cancer and colonisation with coagulase-positive *Staphylococcus aureus* in immunocompetent individuals [5, 6, 10, 53]. By contrast, we found almost no incidence of *S. aureus* in the lesional skin of immunosuppressed subjects, and instead SCC were dominated by CoNS, in particular *S. epidermidis*. Though common skin commensals, CoNS are opportunistic pathogens implicated to cause acute and invasive infections. CoNS-associated disease is frequently reported in transplant patients [56], suggesting immunosuppressed individuals are generally susceptible to overgrowth by commensal microbiota. A study in primary immunodeficiency patients with atopic dermatitis-like eczema, a disease typically characterised by high *S. aureus* abundance, likewise found *S. epidermidis* to be significantly enriched on multiple skin sites in this immunosuppressed cohort, with a decreased proportion of *S. aureus* compared to immunocompetent atopic dermatitis patients [57]. The authors hypothesised that the low abundance of *S. aureus* is caused by antibiotic prophylaxis and therapy that is frequently given to immunodeficient patients, which is often ineffective against *S. epidermidis*. In line with this, we show that a high proportion of our *S. epidermidis* clinical isolates carry genes that mediate resistance to several antibiotics, which may contribute to the dominance of this species in our cohort. Further, *S. epidermidis* may be actively inhibiting *S. aureus* via secretion of a serine protease, previously identified to inhibit *S. aureus* colonisation and biofilm construction [51, 58]. We showed this serine protease to be encoded by all SCC-associated *S. epidermidis* isolates and further confirmed inhibitory activity in in vitro competition experiments.

Interestingly, the *S. epidermidis*-derived DNA polymerase inhibitor 6-HAP was recently demonstrated to protect against skin cancer [31]. As we found *S. epidermidis* overpopulated SCC, one would not expect that these isolates express protective 6-HAP. Indeed, our SCC-associated *S. epidermidis* lacked 6-HAP biosynthesis genes. In fact, the absence of these genes among the broader selection of publicly available genomes suggests that 6-HAP production is uncommon in *S. epidermidis* and that the observed protective phenotype is an exception rather than a rule.

Altogether, the observed expansion of *S. epidermidis* populations in SCC of transplant recipients may indicate that the proliferation of this normally benign commensal species is simply a consequence of an impaired immune system and decreased skin immunosurveillance, combined with survival advantage due to antibiotic resistance and active inhibition of other species. On the other hand, *S. epidermidis* lacking 6-HAP genes may actively promote SCC progression in a dysbiotic state. For instance, the uncontrolled growth of staphylococci may contribute to malignant progression from AK to SCC as microbial dysbiosis and high bacterial loads can trigger recurrent or lasting pro-inflammatory host responses. Chronic inflammation is a well-established promoter of cancer [59] and thus it is plausible that adverse microbial shifts in PDS result in enduring inflammation, which in turn favours malignant transformation of skin epithelial cells.

Findings in the present study should encourage further investigations into a direct causative role for *S. epidermidis* and/or *S. aureus* in the development of SCC. Furthermore, the exploration of the differences in the microbial landscape between intraepidermal and invasive SCC could be of interest. Due to a limited sample size, these two types of SCC were grouped in our study. Low sample numbers also did not permit correlative analyses of pathophysiological features of SCC, immunosuppressive medications, hygiene and skin care routine, and gender. For instance, differences in male and female skin microbiome profiles might be expected due to a number of physiological and behavioural factors, such as differences in hormones, perspiration rate, skin surface pH, and the use of skin care products [60, 61].

As microbial dysbiosis appears to be a common feature of AK and SCC lesions, this study highlights the importance of monitoring the skin microbiome of individuals with severely PDS, particularly in immunosuppressed individuals. Taking measures to re-establish symbiosis may be beneficial to prevent potential complications associated with high microbial load and dysbiosis, such as serious infections and a possible increased susceptibility to malignant transformation of the skin.

## Supplementary information

Supplementary material

Supplementary Table S3

Supplementary Table S4

Supplementary Table S5

Supplementary Table S6

Supplementary Table S7

Supplementary Table S9

## Data Availability

ASV counts and associated metadata for all samples has been provided in the supplementary material (Supplementary Table S4). Amplicon and genome sequencing data have been deposited in the NCBI Sequence Read Archive (https://www.ncbi.nlm.nih.gov/sra) under the Bioprojects PRJNA649052 (isolates from transplant recipients) and PRJNA754839 (isolates from immunocompetent subjects). R code produced for this study has been made available at https://github.com/julianzaugg/Skin_microbiome_SCC_OTR_rRNA_2021.
