# Supplementary material for: Changes in the skin microbiome associated with squamous cell carcinoma in transplant recipients
Source: ISME Commun. 2022 Feb 1;2:13. doi: 10.1038/s43705-022-00095-7 (PMC9723734; doi:10.1038/s43705-022-00095-7)
Supplement: Supplementary file 1 — Supplementary material [file 43705_2022_95_MOESM1_ESM.docx]

# Supplementary Materials and Methods

Ethics, subjects and sampling

32 organ transplant recipients receiving long-term immunosuppressive medication were recruited at the renal and liver transplant clinic of the Princess Alexandra Hospital (Brisbane, Australia) under an ethically approved protocol (HREC/11/QPAH/477). Enrolled were male and female subjects between the ages of 44 and 80 who were at least one year post-transplant and presenting with actinic keratoses (AK) and/or intraepidermal/invasive squamous cell carcinomas (SCC) (subject demographics in **Supplementary Table S1**; clinical and histopathological features of sampled AK/SCC in **Supplementary Figure S1**). Details on hygiene and skin care routines, use of sunscreen, and topical and systemic medications were recorded for each subject. Exclusion criteria for subjects were the presence of chronic skin disorders, and current or prior use (in the preceding three months) of antibiotics and/or topical agents to treat AK (e.g. imiquimod, diclofenac). Skin swab samples were collected from photo-damaged skin (PDS) and AK on the upper limb (predominantly forearm). Additionally, swabs from SCC were collected from the forearms and other body sites, as well as from matching perilesional control skin 2-3cm adjacent to the sampled SCC (SCC_PL). Of the 32 recruited subjects, nine were age-matched transplant recipients without AK / SCC and no or only mild photo-damage. Skin swabs from the forearms of these subjects served as normal skin controls (NS).

The sampling procedure was standardised. Sterile swabs were first dipped into sterile 0.15 M sodium chloride solution and then firmly rotated over a ~1.5cm^2^ sampling area for 30 seconds. Swab heads were collected in sterile glycerol-saline solution and DNA extraction buffer (MO BIO PowerSoil DNA isolation kit). Both were stored immediately at -80°C until further processing. To account for potential microbial contaminants introduced during sampling and processing, negative control swabs not brought into contact with skin were collected in each sampling session and processed identically to skin sample swabs. In total, 216 swab samples from transplant recipients were processed for SSU rRNA gene amplicon sequencing.

Small subunit ribosomal RNA (SSU rRNA) gene amplicon data from our previous study(1) on AK/SCC skin microbiomes from immunocompetent subjects was used for comparative analyses. Details on study design and demographics for this cohort can be found in Wood *et al*. (2018)(1) and in **Supplementary Table S2**. In brief, middle-aged, immunocompetent Caucasian men with severely photo-damaged skin and a history of skin cancer were enrolled at the Princess Alexandra dermatology clinic in Brisbane. AK and non-malignant photo-damaged skin of the forearm were sampled longitudinally for six months. SCC and matching SCC_PL controls from the forearm and any other body site were sampled on a single occasion. The approximately 1,000 swab samples collected from the immunocompetent subjects were reduced to 257 samples (one sample per visit per subject, including 33 negative controls from the full data set) to match the cross-sectional study design of the present study.

In both cohorts, suspected SCC were excised after swabbing and the diagnosis confirmed via histopathology. Diagnosed AK were not confirmed via histopathology as, in the case of the immunocompetent longitudinal study, the lesions were to be swabbed repeatedly over time, and more generally, it was not feasible to request a recruited subject to undergo many excisions (up to 10 AK sampled per subject).

SSU rRNA gene amplicon profiling

To extract microbial DNA, the PowerSoil DNA isolation kit (Qiagen) was used according to the manufacturer’s protocol with small adjustments. After addition of buffer C1, an extra incubation of 10 minutes at 65°C was incorporated. In the final step, DNA was eluted into 50 μL buffer C6. For SSU sequencing, the DNA was further processed as described in our previous publication(1). In brief, the SSU RNA genes were PCR amplified using a universally conserved primer pair (926F and 1392R) with sequence adapters and libraries generated. PCR amplicons were purified, indexed with barcodes, and sequenced on the MiSeq system (Illumina).

Isolation and genome sequencing of staphylococci

Viable microbes sampled by swabbing were preserved in glycerol-saline solution at -80°C. SCC samples (n = 24) and their matching perilesional controls from organ transplant recipients were thawed, vortexed and plated onto staphylococci-selective mannitol salt agar. After 48 hours of growth at 37°C under 5% CO_2_, the colony numbers on each agar plate were assessed. A representative sample (on average five) of each colony type (based on colour, size, morphology, ability to ferment mannitol) from each SCC sample were picked and streaked onto fresh mannitol salt agar. After 24 hours, a single colony was transferred into tryptic soy broth and grown overnight. The bacterial cells were harvested by centrifugation, resuspended in 200 μL DNA extraction buffer and stored at -80°C until further processing for draft genome sequencing.

To extract DNA from staphylococci isolates for genome sequencing, cell collections were thawed and added to 300 mg of silica beads (0.1 mm diameter; BioSpec #11079101) and 1.2 mL lysis buffer (Perkin Elmer CMG-1076). Samples were vortexed, processed on the MoBio Powerlyzer for 5 minutes at 2000 rpm and then centrifuged at 10000 x g for 1 minute. 30 μL of Proteinase K (Perkin Elmer, CMG-820) was added, followed by vortexing. The samples were then incubated while shaking at 70°C for 10 minutes, followed by an incubation at 95°C for 5 minutes and centrifugation at 10000 x g for 1 minute. DNA purification was performed in 96-well plates on the Chemagic™ 360 instrument (#2024-0020) according to the manufacturer’s protocol using 800 μL of supernatant and 75 μL of magnetic beads (Perkin Elmer, CMG-1076). DNA was eluted into 75 μL elution buffer (Perkin Elmer, CMG-1076). DNA libraries were prepared with a Nextera Flex library preparation kit (Illumina, #20018705). Library preparation was run on the Mantis Liquid Handler (Formulatrix). Resulting amplified libraries were cleaned-up as per the “Clean Up Libraries” section in the manufacturer’s protocol. Each library was then quantified using the Quant-iT™ dsDNA HS Assay Kit (Invitrogen) and quality assessed via Agilent D1000 HS tapes (#5067-5582) on the TapeStation 4200 (Agilent # G2991AA) as per the manufacturer’s protocol. Nextera DNA Flex libraries were pooled at equimolar amounts of 0.5 nM per library to create a sequencing pool. The library pool was then again quantified in triplicates using the Qubit™ dsDNA HS Assay Kit (Invitrogen). Library quality control was performed using the Agilent D1000 HS tapes (#5067-5582) on the TapeStation 4200 (Agilent # G2991AA). The library was prepared for sequencing on the NextSeq500 (Illumina) using NextSeq 500/550 High Output v2 2 x 150bp paired end chemistry according to manufacturer’s protocol. Approximately 0.5 GB of sequence data was produced per sample.

Quantification of total DNA and bacterial load

A subsample of collected swabs were tested for total DNA content in duplicate using the Quant-iT™ dsDNA Assay Kit (#Q33120) according to standard protocol (ThermoFisher Scientific, Australia).

For quantitative analysis of bacterial load, qPCR was performed on all swab samples using prokaryotic-specific SSU rRNA primers. The PCR was prepared using 5 µL of 2X QuantiNova SYBR Green PCR Kit (Qiagen, Germany), 4 µL of skin swab DNA and 1 µL of primer mix. The 1406F/1525R primer set (0.4 µM) was designed to amplify bacterial and archaeal 16S rRNA genes: 1406F (5’- GYACWCACCGCCCGT-3’) and 1525R (5’– AAGGAGGTGWTCCARCC-3’) and samples were run in triplicate for each sample. The PCR was conducted on the ViiA7 platform (Applied Biosystems, USA) using the following cycling conditions: 2 minutes at 95°C and 40 cycles of 15 seconds at 95°C, followed by 20 seconds at 55°C and 30 seconds at 72°C. The quantification standard was the same as described in detail in the next section for the *Staphylococcus* qPCR. 16S SSU rRNA copy numbers were then calculated using QuantStudio Real-Time PCR Software v1.3.

*Staphylococcus* genus and *Staphylococcus aureus* duplex qPCR

To estimate the abundance of *Staphylococcus* spp. and *S. aureus* in skin swabs, a duplex real-time PCR assay was adapted from Kilic *et al*.(2). This probe-based PCR assay targets *tuf*, a gene that allows detection of staphylococci at the genus level, and the *nuc* gene, which can distinguish *S. aureus* from other *Staphylococcus* spp. The primer and probe sequences were as follows: Nuc-P1: 5′-gttgcttagtgttaactttagttgta-3′, Nuc-P2: 5′-aatgtcgcaggttctttatgtaattt-3′, Nuc-Probe: 5’-FAM-AAGTCTAAG/ZEN/TAGCTCAGCAAATGCA-IABkFQ-3’ Tuf-P1: 5′-aaacaactgttactggtgtagaaatg-3′, Tuf-P2: 5′-agtacggaaatagaattgtg-3′, Tuf-Probe: 5’-HEX-TCCGTAAATTATTAGACTACGCTGAAGC-BHQ1-3’. Note that a modified version of the Tuf-P1 primer (Tuf-P1-Sa_mod: 5’- aaacaactgttacAggtgtTgaaatg-3’) was also included in the assay to account for conserved variations identified in the sequenced *S. aureus* genomes (see *Isolation and genome sequencing of staphylococci*). The reaction mix consisted of QIAGEN QuantiNova Multiplex PCR mix (QIAGEN, Germany), 0.05 µL QuantiNova ROX reference dye, 4.0 pmol of each Nuc and Tuf-P2 primer, 2.7 pmol of Tuf-P1 primer, 1.3 pmol of Tuf-P1-Sa_mod primer, 1.6 pmol of Nuc probe, 3.2 pmol of Tuf probe, and 2 µl of template in a final 10 µl reaction volume. Five replicates across two runs were conducted on Viia7 instruments (Applied Biosystems, Australia) using the following cycling conditions: 95°C for 2 minutes, and 40 cycles of 95°C for 15 seconds and 60°C for 1 minute. One of the WGS-confirmed *S. aureus* isolates (see *Isolation and genome sequencing of staphylococci*) was used to produce a quantification standard. *S. aureus* culture suspension was pelleted and washed with PBS three times followed by treatment with PMAxx (Biotium, USA) prior to nucleic acid extraction and total dsDNA quantification in triplicate with the Qubit HS dsDNA kit (ThermoFisher Scientific, Australia). Six-point triplicate serial 10-fold dilutions of the *S. aureus* extract were used within each PCR plate to create standard curves for quantification. Genome copy equivalents (Geq/µl) were calculated by dividing the extracts’ mass by the mass of a representative *S. aureus* genome (Genbank assembly accession GCA_000189435.3, <https://www.ncbi.nlm.nih.gov/assembly/GCA_000189435.3/>). The standard curve PCR efficiency and R2 were 95.55% and 0.998 for *S. aureus*, and 92.05% and 0.997 for the *Staphylococcus* spp. assay. The PCR assay’s limit of reliable detection was determined from 20-replicate 2-fold dilutions and defined as the lowest concentration able to be detected in 95% of replicates(3). The limit of quantification was defined as the lowest standard concentration with < 35% coefficient of variation(3). The limits of quantification for *S. aureus* and *Staphylococcus* spp. were identified as 34.7 mean genome equivalents per µL (Geq/µL) and 43.6 Geq/µl, respectively, and the limit of reliable detection was 21 Geq/µl for both gene targets. *Staphylococcus* bacterial loads for a number of samples did not reach the conservative limit of quantification and as a result, the absolute abundance calculations may have a margin of error within them. Despite this limitation, the change in magnitude in the biological signal between sample type groupings allowed for confident analyses. GraphPad Prism (ver. 8.3.1) was used for statistics and visualisation of the *Staphylococcus* genus and *Staphylococcus aureus* qPCR data.

Deferred growth inhibition (competition) assay

Bacterial competition assays were carried out to determine whether *S. epidermidis* from transplant recipients can actively inhibit the growth of *S. aureus*. The inhibitory activity of twelve SCC-associated *S. epidermidis* isolates, each from a different transplant recipient, was tested against six genetically distinct *S. aureus* isolates, including one SCC-derived isolate from a transplant recipient, and five *S. aureus* isolated from AK or SCC from immunocompetent individuals. Additionally, *S. epidermidis* (Winslow and Winslow) Evans ATCC14990 and the methicillin-resistant *S. aureus* USA300 were included as reference strains. Isolates were streaked onto mannitol salt agar from glycerol stocks and incubated at 37°C. After 48 hours, sterile tryptic soy broth (TSB) was inoculated with a single colony of *S. epidermidis* and incubated overnight at 37°C shaking at 250 rpm. The next morning, the OD_600_ was measured, and each *S. epidermidis* culture was diluted to an OD_600_ 0.1 in 1 ml TSB. Then, 10 ul of *S. epidermidis* dilution was carefully pipetted as a spot culture onto a tryptic soy agar (TSA) plate and moved to the 37°C incubator. The same day, TSB was inoculated with a single colony of *S. aureus* and placed in an orbital shaker set at 37°C and 250 rpm. The following day, the OD_600_ of *S. aureus* cultures were assessed, and each culture diluted to an OD_600_ of 0.07 in 50 ml TSA (0.6% agar). The diluted *S. aureus* TSA was then carefully pipetted to cover the agar surface of the plates prepared the previous day containing *S. epidermidis* spot culture (3 ml *S. aureus* TSA per plate). Once the *S. aureus* agar settled, the plates were returned to the 37°C incubator. After 24 hours, photographs of the plates including a scale were taken and the growth inhibition zone (visible zone of no or reduced growth of *S. aureus*) was measured in millimetres using ImageJ. At least three replicates of each *S. aureus*-*S. epidermidis* combination were obtained.

Data availability

Amplicon and genome sequencing data have been deposited in the NCBI Sequence Read Archive (<https://www.ncbi.nlm.nih.gov/sra>) under the Bioprojects PRJNA649052 (isolates from transplant recipients) and PRJNA754839 (isolates from immunocompetent subjects). R code produced for this study has been made available at <https://github.com/julianzaugg/Skin_microbiome_SCC_OTR_rRNA_2021>.

# References

1. Wood DL, Lachner N, Tan J-M, Tang S, Angel N, Laino A, et al. A natural history of actinic keratosis and cutaneous squamous cell carcinoma microbiomes. MBio. 2018;9(5).

2. Kilic A, Muldrew KL, Tang Y-W, Basustaoglu AC. Triplex real-time polymerase chain reaction assay for simultaneous detection of Staphylococcus aureus and coagulase-negative staphylococci and determination of methicillin resistance directly from positive blood culture bottles. Diagnostic microbiology and infectious disease. 2010;66(4):349-55.

3. Forootan A, Sjöback R, Björkman J, Sjögreen B, Linz L, Kubista M. Methods to determine limit of detection and limit of quantification in quantitative real-time PCR (qPCR). Biomolecular detection and quantification. 2017;12:1-6.

# Supplementary Figures


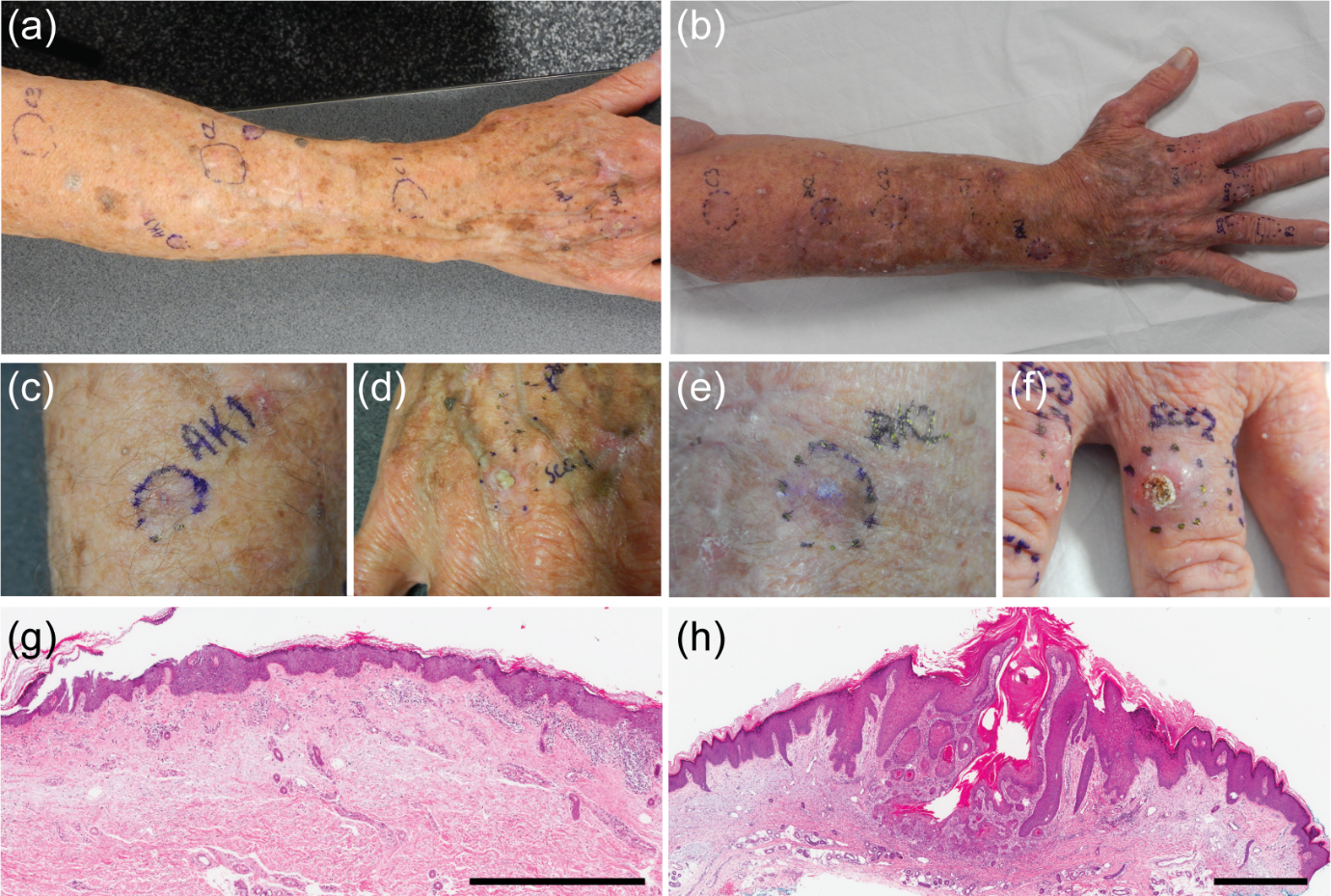


**Figure S1: Clinical and histopathological features of AK and SCC.** Clinical overview of subjects’ forearm with moderate to severe photo-damage (a–b). Close-up clinical photographs of AK (c, e) and SCC (d, f). Histopathology of an AK with atypical keratinocytes within the epidermis (g). Invasive SCC with strands and cords of atypical keratinocytes extending into the dermis (h). Black bar represents 1 mm.

**Figure S2:** Prevalence of amplicon sequence variants (ASVs) across negative control and non-negative control swabs for both organ transplant recipient and immunocompetent cohorts. Likely contaminant ASVs that were more prevalent in negative controls were removed from further analysis.

**Figure S3: Principal component analysis (PCA) visualising the variation in microbial community composition across skin swab samples from both organ transplant recipient and immunocompetent cohorts.** Samples are annotated by **a)** sample type: normal skin (NS), non-malignant photo-damaged skin (PDS), actinic keratosis (AK), intraepidermal and invasive squamous cell carcinoma (SCC) and matching perilesional controls (SCC_PL), and **b)** originating subject.

**Figure S4:** Principal component analysis (PCA) visualising the variation in microbial community composition across skin swab samples from female and male organ transplant recipients.

**Figure S5: Heatmap of mean relative abundances of genera in SCC samples for organ transplant recipient and immunocompetent subjects.** Displayed are the set of top 10 genera by mean relative abundance, calculated across both cohorts and all sample types.

**Figure S6: Heatmap of mean relative abundances of genera in SCC_PL samples for organ transplant recipient and immunocompetent subjects.** Displayed are the set of top 10 genera by mean relative abundance, calculated across both cohorts and all sample types.

**Figure S7: Heatmap of mean relative abundances of genera in AK samples for organ transplant recipient and immunocompetent subjects.** Displayed are the set of top 10 genera by mean relative abundance, calculated across both cohorts and all sample types.

**Figure S8: Heatmap of mean relative abundances of genera in PDS samples for organ transplant recipient and immunocompetent subjects.** Displayed are the set of top 10 genera by mean relative abundance, calculated across both cohorts and all sample types.

**Figure S9: Heatmap of mean relative abundances of genera in NS samples for organ transplant recipient subjects.** Displayed are the set of top 10 genera by mean relative abundance, calculated across both cohorts and all sample types.


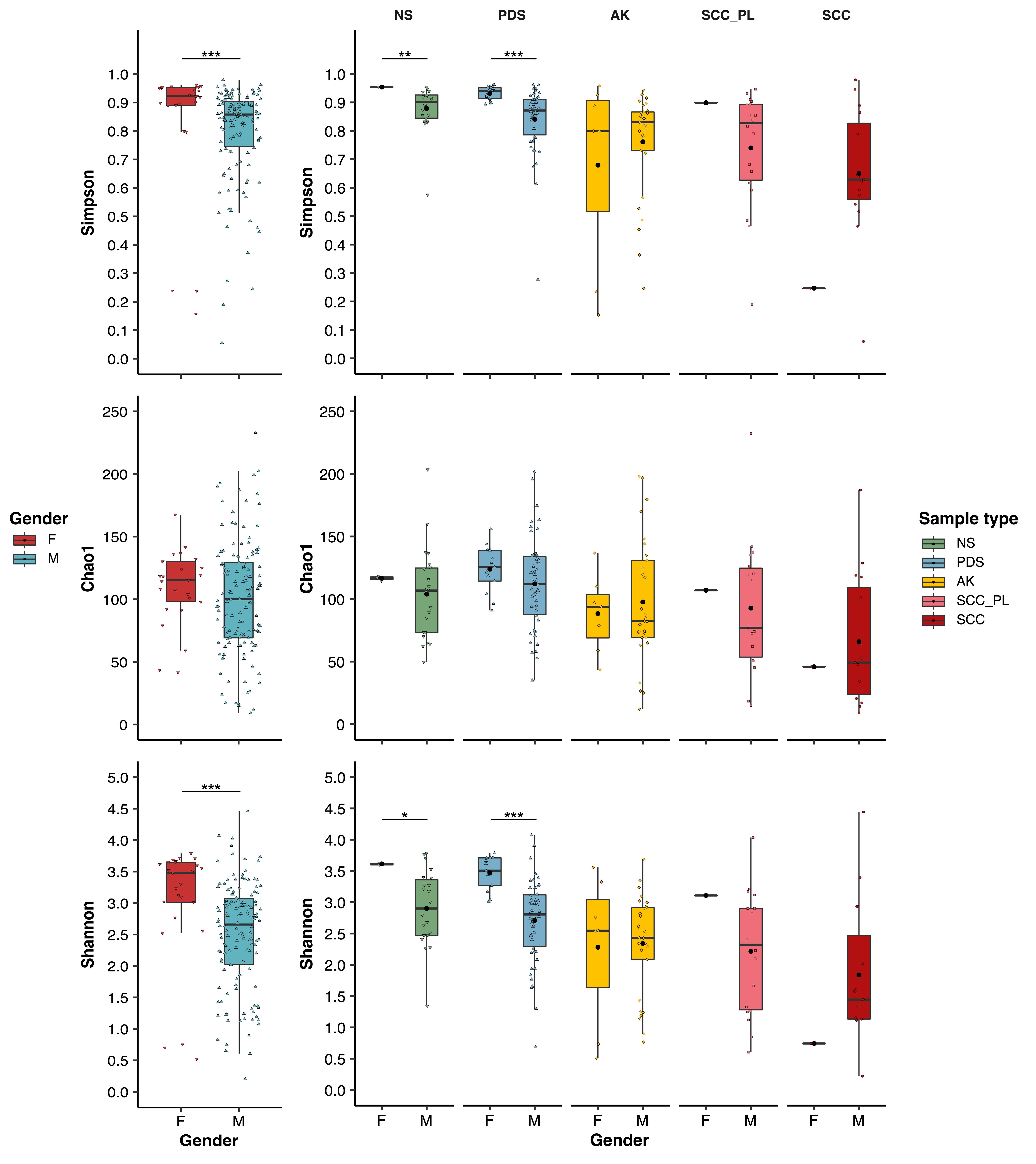


**Figure S10:** **Alpha diversity in control skin, AK and SCC swabs from female and male immunosuppressed individuals.** Tukey style box plots showing genus diversity across genders and sample types for organ transplant recipients. Diversity is represented by three separate measures of alpha diversity; Chao1 (richness), Shannon (diversity) and Simpson (evenness). Bars indicate median +/- 1.5 × interquartile range and the mean diversity for each sample type is indicated by the black dot. Significant differences between sample types are indicated by * = *p* ≤ 0.05, ** = *p* ≤ 0.01 and *** = *p* ≤ 0.001 as calculated via Mann-Whitney U test.

**Figure S11: Relative microbial abundances and absolute bacterial loads in AK, SCC and control skin of immunosuppressed subjects, in response to time (in years) on immunsuppressive medication. (a)** Normalised mean relative abundances of the five most abundant skin microbes in swabs from normal skin (NS), non-malignant photo-damaged skin (PDS), actinic keratosis (AK), intraepidermal and invasive squamous cell carcinoma (SCC) and matching perilesional controls (SCC_PL) from organ transplant recipient subjects, according to duration of immunosuppression (2 to 6 years, 7 to 15 years, and 16 years and higher). Microbes with lower abundances have been combined into ‘Other’ (in grey). Values are calculated from normalised SSU rRNA read counts collapsed to the genus level. **(b)** Tukey style box plots showing bacterial loads in skin swab samples from organ transplant recipient subjects assessed via SSU rRNA qPCR. Bars indicate median +/- 1.5 × interquartile range and the mean is indicated by the black dot. Significant differences between time on immunosuppression medication ranges, as calculated by Dunn’s multiple comparisons tests, are indicated by * = *p* ≤ 0.05, ** = *p* ≤ 0.01, *** = *p* ≤ 0.001 and **** = *p* ≤ 0.0001.

**Figure S12: Bacterial load in skin swab samples from each organ transplant recipient, assessed via SSU rRNA qPCR.**

**Figure S13: Bacterial load in skin swab samples from each immunocompetent subject, assessed via SSU rRNA qPCR.**


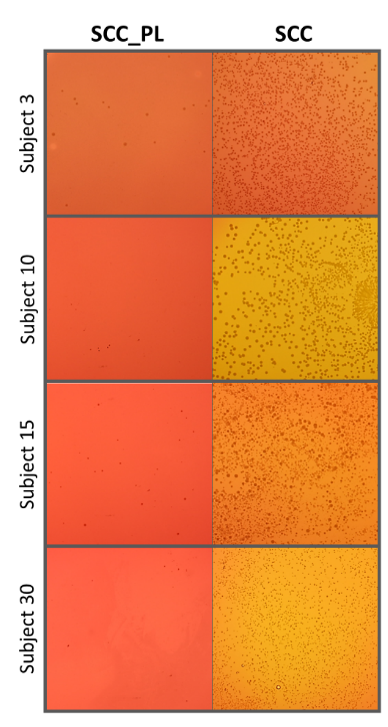
 **a) b)**


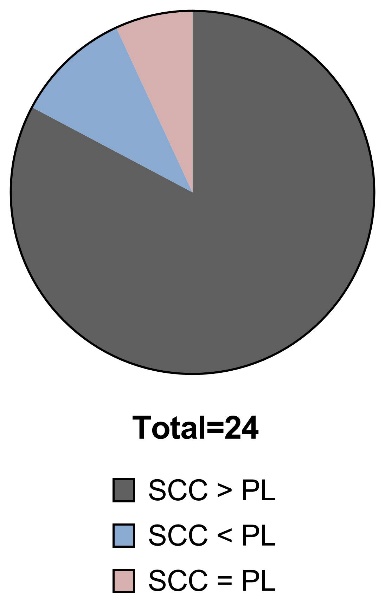


**Figure S14**: **a)** Colony numbers on staphylococci-selective mannitol salt agar 24 hours after plating a standardised amount of skin swab liquid obtained from SCC lesions (right) and matching perilesional control skin (left) from organ transplant recipients. Representative example images from four different subjects (out of 24 plated control-lesion pairs). SCC skin commonly had a highly increased load of staphylococci compared to its site-specific control. **b)** Proportion of cases when the SCC sample either exceeded, had comparable, or a lower staphylococcal load compared to their matching perilesional control sample (out of 24 lesion and control samples; from 14 different organ transplant recipients).

**Figure S15**: Cladogram of maximum likelihood phylogenetic tree for staphylococci isolate and reference strain genomes, built from the core gene alignment from a pangenome analysis. Leaf nodes are coloured by subject, with grey boxes indicating reference strains. Leaf labels are coloured by species, with bolded labels indicating isolate genomes and grey labels indicating reference genomes.

**Figure S16**: Maximum likelihood phylogenetic tree for *S. epidermidis* isolate and reference strain genomes, built from the core-alignment of single nucleotide polymorphisms followed by the removal of recombinant regions. Leaf nodes and bolded labels are coloured by subject, with grey boxes indicating reference strains. The presence (or absence) of genes associated with the resistance to antibiotics, and other virulence factors, is shown in the heatmap panels. Gene labels annotated with *, ** and *** indicate the corresponding gene was derived from CARD, Resfinder or VFDB (otherwise searched for using BLAST).

# Supplementary Tables

**Table S1:** Organ transplant recipient cohort subject demographics.

| **Subject** | **Transplant** | **Transplant Year** | **Year Recruited** | **Length of immunosuppression (years)** | **Gender** | **Age** | **NS** | **PDS** | **AK** | **SCC** | **SCC** | **Location** |
| --- | --- | --- | --- | --- | --- | --- | --- | --- | --- | --- | --- | --- |
| **#** |  |  |  |  |  |  |  |  |  | **-PL** |  | **of SCC** |
| **1** | Heart, | 2003 | Sep-16 | 13 | F | 70 | 0 | 3 | 3 | 0 | 0 | - |
|  | Renal | 2003 |  |  |  |  |  |  |  |  |  |  |
| **2** | Renal | 2007 | Sep-16 | 9 | M | 44 | 0 | 3 | 3 | 1 | 1 | Head and neck |
| **3** | Renal | 2013 | Sep-16 | 3 | M | 57 | 0 | 4 | 4 | 5 | 5 | 3x Arm; 2x Hand |
| **4** | Renal (2x) | 1983, 2006 | Oct-16 | 33 | F | 69 | 0 | 3 | 3 | 0 | 0 | - |
| **5** | Renal | 2006 | Oct-16 | 10 | M | 67 | 0 | 3 | 3 | 0 | 0 | - |
| **6** | Renal | 2011 | Oct-16 | 5 | M | 63 | 0 | 3 | 3 | 0 | 0 | - |
| **7** | Renal | 2010 | Oct-16 | 6 | M | 61 | 3 | 0 | 0 | 0 | 0 | - |
| **8** | Renal (2x) | 1976, 1990 | Oct-16 | 40 | M | 67 | 0 | 3 | 3 | 1 | 1 | Head and neck |
| **9** | Renal | 2014 | Nov-16 | 2 | M | 53 | 3 | 0 | 0 | 0 | 0 | - |
| **10** | Renal | 2007 | Nov-16 | 9 | M | 63 | 3 | 0 | 0 | 0 | 0 | - |
| **11** | Renal | 2012 | Nov-16 | 4 | M | 71 | 0 | 3 | 2 | 0 | 0 | - |
| **12** | Renal | 2006 | Nov-16 | 10 | M | 80 | 0 | 3 | 3 | 2 | 2 | Arm, Lower leg |
| **13** | Renal (2x) | 1983, 2016 | Nov-16 | 33 | F | 56 | 0 | 3 | 3 | 2 | 2 | 2x Hand |
| **14** | Renal | 1996 | Nov-16 | 20 | M | 45 | 0 | 4 | 4 | 0 | 0 | - |
| **15** | Renal (2x) | 1981, 1982 | Nov-16 | 35 | M | 60 | 0 | 3 | 3 | 0 | 1 | Foot |
| **16** | Renal (2x) | 1987, 2001 | Dec-16 | 29 | M | 63 | 0 | 3 | 3 | 1 | 1 | Head and neck |
| **17** | Renal | 2000 | Dec-16 | 16 | M | 60 | 0 | 3 | 0 | 1 | 1 | Head and neck |
| **18** | Renal | 2010 | Dec-16 | 6 | M | 67 | 0 | 3 | 0 | 1 | 1 | Arm |
| **19** | Renal | 2006 | Jan-17 | 11 | M | 64 | 3 | 0 | 0 | 0 | 0 | - |
| **20** | Renal | 2009 | Jan-17 | 8 | M | 54 | 0 | 3 | 0 | 1 | 1 | Head and neck |
| **21** | Renal (2x) | 1988, 2005 | Jan-17 | 29 | M | 46 | 0 | 3 | 3 | 0 | 0 | - |
| **22** | Liver (2x), Renal | 2001, 2011 | Feb-17 | 16 | F | 63 | 0 | 4 | 1 | 0 | 0 | - |
|  |  | 2001 |  |  |  |  |  |  |  |  |  |  |
| **23** | Liver | 1998 | Mar-17 | 19 | M | 59 | 0 | 4 | 1 | 1 | 1 | Arm |
| **24** | Renal | 2014 | Mar-17 | 3 | M | 70 | 3 | 0 | 0 | 0 | 0 | - |
| **25** | Renal | 1988 | Mar-17 | 29 | M | 54 | 3 | 0 | 0 | 0 | 0 | - |
| **26** | Renal | 2000 | Mar-17 | 17 | F | 68 | 0 | 3 | 1 | 1 | 1 | Arm |
| **27** | Renal | 2009 | Apr-17 | 8 | M | 54 | 3 | 0 | 0 | 0 | 0 | - |
| **28** | Renal | 2012 | Apr-17 | 5 | M | 66 | 3 | 0 | 0 | 0 | 0 | - |
| **29** | Renal | 2000 | Apr-17 | 17 | M | 72 | 0 | 3 | 2 | 3 | 3 | Hand, Arm, Head and neck |
| **30** | Renal | 1996 | Jun-17 | 21 | M | 78 | 0 | 3 | 0 | 3 | 3 | Hand, 2xArm |
| **31** | Renal | 2002 | Jun-17 | 15 | F | 64 | 3 | 0 | 0 | 0 | 0 |  |
| **32** | Renal | 1992 | Jul-17 | 25 | M | 54 | 0 | 3 | 3 | 0 | 0 |  |

NS = Normal skin control; PDS = Photo-damaged skin; AK = Actinic keratosis; SCC = cutaneous squamous cell carcinoma; SCC-PL = Squamous cell carcinoma perilesional skin control

**Table S2:** Immunocompetent cohort subject demographics (subset of swab samples from Wood *et al*., 2018).

| **Subject** | **Year Recruited** | **Gender** | **Age** | **PDS** | **AK** | **SCC** | **SCC** | **Location** |
| --- | --- | --- | --- | --- | --- | --- | --- | --- |
| **#** |  |  |  |  |  | **-PL** |  | **of SCC** |
| **1** | Jul-15 | M | 66 | 6 | 12 | 1 | 1 | Head and neck |
|  |  |  |  |  |  |  |  |  |
| **2** | Aug-15 | M | 89 | 6 | 12 | 1 | 2 | Lower leg |
| **4** | Jul-16 | M | 56 | 4 | 4 | 1 | 2 | Lower arm |
| **5** | Jun-15 | M | 76 | 6 | 9 | 1 | 1 | Chest |
| **6** | Mar-16 | M | 77 | 3 | 3 | 2 | 3 | Head and neck,  2x lower arm |
| **7** | Jul-15 | M | 73 | 6 | 12 | 1 | 1 | Lower leg |
| **8** | Aug-15 | M | 78 | 7 | 12 | 6 | 6 | 2x Lower arm, Upper arm, 3x Lower leg |
| **9** | Oct-15 | M | 68 | 6 | 12 | 1 | 1 | Lower leg |
| **10** | Jul-15 | M | 65 | 5 | 12 | 6 | 6 | 5x Arm, 2x Hand |
| **11** | Jul-16 | M | 65 | 6 | 7 | 7 | 8 | 6x Head and neck, 2x Lower arm |
| **12** | Oct-15 | M | 88 | 0 | 0 | 1 | 1 | Head and neck |
| **13** | Nov-15 | M | 72 | 0 | 0 | 1 | 1 | Lower leg |
| **14** | Nov-15 | M | 76 | 0 | 0 | 6 | 6 | Chest, Upper arm, 4x Head and neck |

NS = Normal skin control; PDS = Photo-damaged skin; AK = Actinic keratosis;

SCC = cutaneous squamous cell carcinoma; SCC-PL = Squamous cell carcinoma

perilesional skin control

**Table S3**: Reference genomes and virulence genes used for phylogenetic analyses of isolates (see separate excel spreadsheet)

**Table S4**: ASV feature counts and metadata for all samples (see separate excel spreadsheet)

**Table S5**: PERMANOVA results (see separate excel spreadsheet)

**Table S6**: DESeq results (see separate excel spreadsheet)

**Table S7**: Dunn’s multiple comparisons tests of relative abundance profiles (see separate excel spreadsheet)

**Table S8**: Total DNA concentrations in a subset of swabs collected from different clinicians.

NS = Normal skin control; PDS = Photo-damaged skin; AK = Actinic keratosis; SCC = cutaneous squamous cell carcinoma; SCC-PL = Squamous cell carcinoma perilesional skin control; OTR = Organ transplant recipient cohort; IC = Immunocompetent cohort

**Table S9**: 6-HAP marker results (see separate excel spreadsheet)
